# Traumatic Brain Injury–Related Emergency Department Visits, Hospitalizations, and Deaths — United States, 2007 and 2013

**DOI:** 10.15585/mmwr.ss6609a1

**Published:** 2017-03-17

**Authors:** Christopher A. Taylor, Jeneita M. Bell, Matthew J. Breiding, Likang Xu

**Affiliations:** 1 Division of Unintentional Injury Prevention, National Center for Injury Prevention and Control; 2 Division of Analysis, Research, and Practice Integration, National Center for Injury Prevention and Control

## Abstract

**Problem/Condition:**

Traumatic brain injury (TBI) has short- and long-term adverse clinical outcomes, including death and disability. TBI can be caused by a number of principal mechanisms, including motor-vehicle crashes, falls, and assaults. This report describes the estimated incidence of TBI-related emergency department (ED) visits, hospitalizations, and deaths during 2013 and makes comparisons to similar estimates from 2007.

**Reporting Period:**

2007 and 2013.

**Description of System:**

State-based administrative health care data were used to calculate estimates of TBI-related ED visits and hospitalizations by principal mechanism of injury, age group, sex, and injury intent. Categories of injury intent included unintentional (motor-vehicle crashes, falls, being struck by or against an object, mechanism unspecified), intentional (self-harm and assault/homicide), and undetermined intent. These health records come from the Healthcare Cost and Utilization Project’s National Emergency Department Sample and National Inpatient Sample. TBI-related death analyses used CDC multiple-cause-of-death public-use data files, which contain death certificate data from all 50 states and the District of Columbia.

**Results:**

In 2013, a total of approximately 2.8 million TBI-related ED visits, hospitalizations, and deaths (TBI-EDHDs) occurred in the United States. This consisted of approximately 2.5 million TBI-related ED visits, approximately 282,000 TBI-related hospitalizations, and approximately 56,000 TBI-related deaths. TBIs were diagnosed in nearly 2.8 million (1.9%) of the approximately 149 million total injury- and noninjury-related EDHDs that occurred in the United States during 2013. Rates of TBI-EDHDs varied by age, with the highest rates observed among persons aged ≥75 years (2,232.2 per 100,000 population), 0–4 years (1,591.5), and 15–24 years (1,080.7). Overall, males had higher age-adjusted rates of TBI-EDHDs (959.0) compared with females (810.8) and the most common principal mechanisms of injury for all age groups included falls (413.2, age-adjusted), being struck by or against an object (142.1, age-adjusted), and motor-vehicle crashes (121.7, age-adjusted). The age-adjusted rate of ED visits was higher in 2013 (787.1) versus 2007 (534.4), with fall-related TBIs among persons aged ≥75 years accounting for 17.9% of the increase in the number of TBI-related ED visits. The number and rate of TBI-related hospitalizations also increased among persons aged ≥75 years (from 356.9 in 2007 to 454.4 in 2013), primarily because of falls. Whereas motor-vehicle crashes were the leading cause of TBI-related deaths in 2007 in both number and rate, in 2013, intentional self-harm was the leading cause in number and rate. The overall age-adjusted rate of TBI-related deaths for all ages decreased from 17.9 in 2007 to 17.0 in 2013; however, age-adjusted TBI-related death rates attributable to falls increased from 3.8 in 2007 to 4.5 in 2013, primarily among older adults. Although the age-adjusted rate of TBI-related deaths attributable to motor-vehicle crashes decreased from 5.0 in 2007 to 3.4 in 2013, the age-adjusted rate of TBI-related ED visits attributable to motor-vehicle crashes increased from 83.8 in 2007 to 99.5 in 2013. The age-adjusted rate of TBI-related hospitalizations attributable to motor-vehicle crashes decreased from 23.5 in 2007 to 18.8 in 2013.

**Interpretation:**

Progress has been made to prevent motor-vehicle crashes, resulting in a decrease in the number of TBI-related hospitalizations and deaths from 2007 to 2013. However, during the same time, the number and rate of older adult fall-related TBIs have increased substantially. Although considerable public interest has focused on sports-related concussion in youth, the findings in this report suggest that TBIs attributable to older adult falls, many of which result in hospitalization and death, should receive public health attention.

**Public Health Actions:**

The increase in the number of fall-related TBIs in older adults suggests an urgent need to enhance fall-prevention efforts in that population. Multiple effective interventions have been identified, and CDC has developed the STEADI initiative (Stopping Elderly Accidents Deaths and Injuries) as a comprehensive strategy that incorporates empirically supported clinical guidelines and scientifically tested interventions to help primary care providers address their patients’ fall risk through the identification of modifiable risk factors and implementation of effective interventions (e.g., exercise, medication management, and Vitamin D supplementation).

## Introduction

CDC conducts surveillance of traumatic brain injury (TBI) to understand the public health burden, to monitor trends, and to identify groups at greatest risk for TBI. TBI is a major cause of mortality in the United States, contributing to approximately 30% of all injury-related deaths (*1*). The burden on the health care system is also substantial; in 2010, approximately 2.5 million emergency department (ED) visits, hospitalizations, or deaths were associated with TBI (*2*). From 2001 to 2010, the rates of ED visits increased by 70%, and rates of hospitalization and death increased by 11% and 7%, respectively (*2*).

Historically, CDC’s national estimates of TBI-related hospitalizations and ED visits have been based on data from two sources: the National Hospital Discharge Survey (NHDS) and the National Hospital Ambulatory Medical Care Survey (NHAMCS), respectively. These databases have been useful in identifying broad groups affected by TBI and the most common principal mechanisms of injury resulting in TBI. However, the relatively small sample sizes of these databases have not allowed for the calculation of stable annual estimates by subgroups of interest, such as estimates stratified by both age group and principal mechanism of injury. As a result, pooled years of data were required to achieve stability; for example, data published by CDC in 2010 included estimates of TBI-related medical encounters from NHDS and NHAMCS that were pooled for 2002–2006. This limits the ability of researchers to document changes in TBI incidence over time, especially for subgroups of particular risk (e.g., falls among children and older adults and sports-related TBIs among youth). To improve TBI estimation, CDC identified alternative databases that were large enough to provide annual estimates of TBI within subgroups. CDC uses the Healthcare Cost and Utilization Project (HCUP) databases to estimate the incidence of TBI in the United States (*3*–*5*). The findings in this report can be used by public health officials to understand trends in TBI-related medical events (e.g., emergency department visits, hospitalizations and deaths) and to identify priority areas for prevention programs.

## Methods

To estimate the incidence of TBI-related emergency department (ED) visits, hospitalizations, and deaths (TBI-EDHDs) during 2013 compared to 2007, CDC analyzed the latest data from two HCUP databases and the National Vital Statistics System (NVSS). The findings are reported by principal mechanism of injury, age group, sex, and medical event type (i.e., ED visits, hospitalizations, deaths) for 2013. In addition, estimates from 2007 and 2013 were compared to describe changes in TBI incidence since CDC’s most recent detailed report examining data during 2002–2006 (*1*). Traumatic brain injury-related deaths were analyzed using the multiple-cause-of-death files from the 2007 and 2013 NVSS.

### Data Sources

The HCUP Nationwide Emergency Department Sample (NEDS) and HCUP National Inpatient Sample (NIS) are visit-based databases that contain sample sizes large enough to provide stable annual estimates of TBI for subgroups (*3*,*4*). Previous work has found that the frequencies of TBI-related hospitalizations and ED visits in these HCUP databases are comparable to those observed in NHDS and NHAMCS (*5*). NVSS includes data for all deaths registered in all 50 U.S. states and the District of Columbia (*6*).

Data used to describe TBI-related ED visits were obtained from the 2007 and 2013 NEDS. NEDS is the largest all-payer ED record database in the United States; in 2013, it contained 29,581,718 records representing 134,869,015 ED visits nationally (*4*). NEDS is designed to represent a 20% stratified sample of U.S. hospital-based EDs with records drawn from non-Federal, short-term, general, and other specialty hospitals from participating states (30 states in 2013) (*4*).

TBI-related inpatient hospitalizations were analyzed using data from the 2007 and 2013 NIS. Similar to NEDS, NIS is the largest all-payer inpatient hospitalization database; in 2013, it contained data from 7,119,563 records, representing 35,597,792 hospital discharges nationally (*3*). Throughout the report, NIS discharges are referred to as hospitalizations. The 2013 data represent a 20% sample of hospital discharges from community hospitals, excluding rehabilitation and long-term acute care facilities (*3*).

Records from both NEDS and NIS do not include individual patient identifiers and, unlike the mortality data, might contain more than one record per person (i.e., multiple ED visits for the same injury or multiple medical event types for different injuries). Certain records were excluded to reduce the possibility of counting multiple encounters for the same injury. In-hospital deaths were excluded from the hospitalization counts (because these would be included in the mortality data), as were hospital transfers and hospital admissions that occurred directly from the ED (because each would be included in the hospitalization data).

### Surveillance Definition

For TBI-related ED visits and hospitalizations, cases were identified using codes from the International Classification of Diseases, Ninth Edition, Clinical Modification (ICD-9-CM) using an established definition (*7*). Records were included and classified as TBI-related if any of the following ICD-9-CM codes were included in the record as a diagnosis code, regardless of its position (i.e., both primary and secondary diagnoses were included):

800: fracture of vault of skull;
801: fracture of the base of skull;
803: other and unqualified skull fractures;
804: multiple fractures involving skull or face with other bones;
850: concussion;
851: cerebral laceration and contusion;
852: subarachnoid, subdural, and extradural hemorrhage, following injury;
853: other and unspecified intracranial hemorrhage following injury;
854.0, 854.1: intracranial injury of other and unspecified nature;
950.1–950.3: injury to the optic nerve and pathways;
959.01: head injury, unspecified; and
995.55: shaken infant syndrome.

For TBI-related deaths, cases were included if the record contained a TBI-related code from the International Classification Diseases, Tenth Revision (ICD-10) in any position of the NVSS mortality record. This coding definition has been used previously in the identification of TBI-related mortality records (*1*). These codes included:

S01: open wound of the head;
S02.0, S02.1, S02.3, S02.7–S02.9: fracture of the skull and facial bones;
S04.0: injury to optic nerve and pathways;
S06: intracranial injury;
S07.0, S07.1, S07.8, S07.9: crushing injury of head;
S09.7–S09.9: other unspecified injuries of head;
T01.0: open wounds involving head with neck;
T02.0: fractures involving head with neck;
T04.0: crushing injuries involving head with neck;
T06.0: injuries of brain and cranial nerves with injuries of nerves and spinal cord at neck level; and
T90.1, T90.2, T90.4, T90.5, T90.8, T90.9: sequelae of injuries of head.

### Variables

Data were stratified by age, sex, principal mechanism of injury, and injury intent. Age groups included ages 0–4, 5–14, 15–24, 25–34, 35–44, 45–54, 55–64, 65–74, and ≥75 years. Principal mechanisms of injury included motor-vehicle crashes, falls, being struck by or against an object, mechanism unspecified, and other. Categories of injury intent included unintentional (motor-vehicle crashes, falls, being struck by or against an object, mechanism unspecified), intentional (self-harm and assault/homicide), and undetermined intent. Estimates of intentional self-harm were suppressed for the 0–4 and 5–14 year age groups because it is unclear whether children aged <10 years are truly able to form suicidal intent (*8*). For ED visits and hospitalizations, these categories were based on CDC’s recommended framework for grouping external cause-of-injury codes (E-codes) using ICD-9-CM (*9*). Up to four E-codes can be recorded per ED visit or hospitalization in NEDS or NIS. The first-listed E-code should correspond to the most serious diagnosis assigned (*10*). Therefore, only the first-listed valid E-code was used in this analysis, and this assigned code was assumed to be the principal mechanism of injury for the TBI. Valid injury mechanism E-codes (E800–E966 and E968–E999) are those that are correct in syntax (i.e., they exist as stated within ICD-9-CM) and correspond to a principal mechanism of injury rather than an activity (code E000), place of occurrence (E001–E030), or perpetrator of abuse (E967). Principal mechanism of injury and injury intent for TBI-related deaths were categorized on the basis of the CDC-recommended external cause of injury mortality matrix for ICD-10 (*11*).

### Statistical Analysis

For ED visits and hospitalizations, data were weighted to provide national estimates of annual numbers and rates per 100,000 population. These rates and 95% confidence intervals around each rate were based on U.S. bridged-race population estimates of the resident population released and maintained by CDC for individual years (*12*). Age-adjusted rates were standardized to the U.S. census population estimates for 2000 by the direct method (*1*). Estimates were considered unreliable and not reported if the relative standard error was >30% or the standard error = 0. Chi-square or t-tests were used to analyze between-group differences for number and rates, respectively, of TBI-related ED visits, hospitalizations, and deaths. Only selected comparisons were tested for statistical significance. Differences with p values <0.05 were considered statistically significant. All statistical analyses were performed using SAS Systems for Windows, version 9.3 (SAS Institute, Cary, North Carolina).

The sampling frame for NIS data changed in 2012. Before 2012, NIS data were derived from a sample of hospitals with all discharges included. Beginning in 2012, NIS data were based on a sample of discharges from all NIS-participating hospitals. Revised weights for trend analyses using 2011 NIS data and earlier were used to calculate estimates comparable to the new design in accordance with NIS guidance (*13*).

## Results

In 2013, a total of 2.8 million TBI-EDHDs occurred in the United States (Table 1). This consisted of approximately 2.5 million TBI-related ED visits, approximately 282,000 TBI-related hospitalizations, and approximately 56,000 TBI-related deaths. Of the 149 million total injury and noninjury-related EDHDs that occurred in the United States in 2013, TBIs were diagnosed in 1.9% of these. The proportion of TBI-related injuries accounted for approximately one of every 50 ED visits (2.2%). TBI-related deaths accounted for 2.2% of all deaths in the United States.

**TABLE 1:** Estimated number of emergency department visits,* hospitalizations,^†^ and deaths,^§^ and the number and proportion that were attributable to injury and traumatic brain injury — United States, 2013

Event	Total	Injuries		TBIs		
	No.	No.	% of total events	No.	% of injuries	% of total events
Emergency department visit	**113,370,448**	24,597,345	21.7	2,460,420	10.0	2.2
Hospitalization	**33,288,183**	1,554,104	4.7	281,610	18.1	0.9
Death	**2,596,993**	192,945	7.4	55,927	29.9	2.2
**Total**	**149,255,624**	**26,344,394**	**17.7**	**2,797,957**	**10.6**	**1.9**

**Sources: **For emergency department visits, Healthcare Cost and Utilization Project’s (HCUP) Nationwide Emergency Department Sample; for hospitalizations, HCUP’s National Inpatient Sample; for deaths, CDC’s National Vital Statistics System.

**Abbreviation:** TBIs = traumatic brain injuries.

*Persons who were hospitalized, died, or transferred to another facility were excluded.

^†^In-hospital deaths and patients who transferred from another hospital were excluded.

^§^Record-Axis Condition codes were used (usually included both Part I and Part II of Entity-Axis Condition codes).

### TBI-Related ED Visits, Hospitalizations, and Deaths by Age Group

Rates of TBI-EDHD varied by age, with the highest rates observed among those aged ≥75 years (2,232.2 per 100,000 population), 0–4 years (1,591.5), and 15–24 years (1,080.7) (Table 2). With respect to TBI-related ED visits only, the age groups with the highest rates were those aged ≥75 years (1,701.7), 0–4 years (1,541.1), and 15–24 years (1,001.9). Rates of TBI-related hospitalizations and deaths were highest among the oldest age groups. For hospitalizations, the age groups with the highest rates were those aged ≥75 years (454.4), 65–74 years (139.4), and 55–64 years (86.0). The same pattern was observed for TBI-related deaths with the highest rates found among those aged ≥75 years (76.1), 65–74 years (24.3), and 55–64 years (18.8). In terms of overall numbers, persons aged 15–24 years accounted for 17.9% of all TBI-related ED visits, more than any other age group. Those aged ≥75 years comprised the largest proportion of both TBI-related hospitalizations (31.4%) and deaths (26.5%).

**TABLE 2:** Estimated number* and rate of traumatic brain injury–related emergency department visits, hospitalizations, and deaths, by age group — United States, 2007 and 2013

Age group (yrs)	Year	Emergency department visits^†^		Hospitalizations^§^		Deaths^¶^		Total	
		No.	Rate** (95% CI)	No.	Rate (95% CI)	No.	Rate (95% CI)	No.	Rate (95% CI)
0–4	2007	234,003	1,118.5 (996.3–1,240.7)	10,935	52.3 (39.2–65.4)	978	4.7 (4.4–5.0)	**245,916**	**1,175.4 (1,052.7–1,298.1)**
	2013	305,969	1,541.1 (1,389.9–1,692.3)	9,250	46.6 (40.2–53.0)	760	3.8 (3.6–4.1)	**315,979**	**1,591.5 (1,440.4–1,742.6)**
5–14	2007	227,848	564.5 (515.5–613.6)	12,475	30.9 (24.2–37.6)	975	2.4 (2.3–2.6)	**241,298**	**597.9 (548.4–647.3)**
	2013	335,966	814.8 (744.3–885.4)	8,680	21.1 (18.2–23.9)	724	1.8 (1.6–1.9)	**345,370**	**837.6 (767.1–908.1)**
15–24	2007	340,846	792.8 (749.5–836.1)	40,862	95.0 (80.3–109.8)	8,550	19.9 (19.5–20.3)	**390,258**	**907.7 (862.1–953.4)**
	2013	441,187	1,001.9 (951.2–1,052.7)	28,330	64.3 (59.0–69.6)	6,359	14.4 (14.1–14.8)	**475,876**	**1,080.7 (1,029.7–1,131.6)**
25–34	2007	190,565	473.4 (443.9–502.9)	28,067	69.7 (59.2–80.3)	6,862	17.0 (16.6–17.5)	**225,494**	**560.2 (528.9–591.4)**
	2013	279,583	651.7 (614.2–689.3)	25,990	60.6 (55.7–65.4)	6,605	15.4 (15.0–15.8)	**312,178**	**727.7 (689.9–765.5)**
35–44	2007	156,763	367.1 (344.4–389.8)	27,074	63.4 (54.0–72.8)	6,650	15.6 (15.2–15.9)	**190,487**	**446.0 (421.5–470.6)**
	2013	206,628	509.7 (481.1–538.2)	20,545	50.7 (46.8–54.5)	5,688	14.0 (13.7–14.4)	**232,861**	**574.4 (545.6–603.1)**
45–54	2007	140,709	322.8 (303.0–342.7)	31,795	73.0 (63.3–82.6)	7,774	17.8 (17.4–18.2)	**180,278**	**413.6 (391.6–435.7)**
	2013	218,968	499.4 (471.6–527.2)	31,360	71.5 (66.8–76.2)	7,440	17.0 (16.6–17.4)	**257,768**	**587.9 (559.8–616.1)**
55–64	2007	90,074	275.9 (259.6–292.2)	25,590	78.4 (69.7–87.1)	5,917	18.1 (17.7–18.6)	**121,581**	**372.4 (353.9–390.8)**
	2013	186,186	473.2 (447.7–498.8)	33,815	86.0 (81.1–90.8)	7,401	18.8 (18.4–19.2)	**227,402**	**578.0 (552.0–604.0)**
65–74	2007	66,740	343.2 (321.8–364.6)	24,597	126.5 (115.5–137.5)	4,742	24.4 (23.7–25.1)	**96,079**	**494.1 (470.0–518.1)**
	2013	154,668	613.3 (577.1–649.4)	35,160	139.4 (132.9–145.9)	6,137	24.3 (23.7–24.9)	**195,965**	**777.0 (740.3–813.7)**
≥75	2007	155,574	841.9 (778.1–905.8)	65,955	356.9 (331.1–382.8)	12,251	66.3 (65.1–67.5)	**233,780**	**1,265.2 (1,196.4–1,333.9)**
	2013	331,122	1,701.7 (1,578.2–1,825.2)	88,425	454.4 (436.7–472.1)	14,806	76.1 (74.9–77.3)	**434,353**	**2,232.2 (2,107.7–2,356.8)**
**Total**	**2007**	**1,603,124**	**531.9 (502.7–561.1)**	**267,350**	**88.7 (79.4–98.0)**	**54,699**	**18.1 (18.0–18.3)**	**1,925,173**	**638.8 (608.1–669.4)**
	**2013**	**2,460,278**	**777.5 (736.8–818.2)**	**281,555**	**89.0 (84.8–93.2)**	**55,920**	**17.7 (17.5–17.8)**	**2,797,753**	**884.2 (843.3–925.0)**
**Adjusted^††^**	**2007**	**534.4 (519.8–549.0)**		**87.6 (83.7–91.6)**		**17.9 (17.7–18.0)**		**639.9 (624.7–655.0)**	
	**2013**	**787.1 (767.4–806.9)^§§^**		**85.4 (83.5–87.3)**		**17.0 (16.9–17.2)^§§^**		**889.6 (869.7–909.4)**	

**Sources: **For emergency department visits, Healthcare Cost and Utilization Project’s (HCUP) Nationwide Emergency Department Sample; for hospitalizations, HCUP’s National Sample; for deaths, CDC’s National Vital Statistics System.

**Abbreviation: **CI = confidence interval**.**

*Visits with missing age were excluded. Numbers subject to rounding error.

^†^Persons who were hospitalized, died, or transferred to another facility were excluded.

^§^In-hospital deaths and patients who transferred from another hospital were excluded.

^¶^Record-Axis Condition codes were used (usually included both Part I and Part II of Entity-Axis Condition codes).

**Per 100,000 population.

^††^Age-adjusted to the 2000 U.S. standard population.

^§§^Significant difference in rate compared to 2007 based on t-test (p<0.05). Only selected comparisons were tested for statistical significance.

### TBI-Related ED Visits, Hospitalizations, and Deaths by Age Group and Principal Mechanism of Injury

Rates of TBI-EDHDs varied by principal mechanism of injury, and by age group within principal mechanism of injury (Table 3). Overall, the most common principal mechanisms of injury were falls (413.2 per 100,000 population, age-adjusted), being struck by or against an object (142.1, age-adjusted), and motor-vehicle crashes (121.7, age-adjusted). These principal mechanisms represented 47.2%, 15.4%, and 13.7% of all TBI-EDHDs, respectively. Examining each principal mechanism by age group reveals a different pattern. Those aged ≥75 years had the highest rate of fall-related TBI-EDHDs (1,859.0), followed by 0–4 years (1,119.3), and 65–74 years (539.8). For TBI-EDHDs attributable to being struck by or against an object, the age groups with the highest rate include those aged 5–14 years (291.9), 0–4 years (262.7), and 15–24 years (243.3). Finally, for TBI-EDHDs attributable to motor-vehicle crashes, those aged 15–24 years (258.3), 25–34 years (182.9), and 35–44 years (126.5) had the highest rates.

**TABLE 3:** Estimated number* and rate of traumatic brain injury–related emergency department visits,^†^ hospitalizations,**^§^** and deaths,^¶^ by age group and mechanism of injury — United States, 2013

Age group (yrs)	Unintentional motor-vehicle crashes		Unintentional falls**		Unintentionally struck by or against an object		Other unintentional injury, mechanism unspecified^††^		Intentional self-harm		Assault or homicide		Other or no mechanism specified^§§^	
	No.	Rate^¶¶^ (95% CI)	No.	Rate (95% CI)	No.	Rate (95% CI)	No.	Rate (95% CI)	No.	Rate (95% CI)	No.	Rate (95% CI)	No.	Rate (95% CI)
0–4	6,335	31.9 (28.7–35.2)	222,233	1,119.3 (1,007.8–1,230.9)	52,148	262.7 (235.3–290.0)	13,485	67.9 (56.6–79.3)	—***	—	1,979	10.0 (8.7–11.3)	19,756	99.5 (54.9–144.1)
5–14	21,308	51.7 (47.4–56.0)	132,190	320.6 (291.2–350.0)	120,375	291.9 (264.7–319.2)	33,623	81.5 (74.4–88.6)	—	—	10,109	24.5 (22.0–27.0)	27,414	66.5 (41.6–91.4)
15–24	113,743	258.3 (243.2–273.4)	100,364	227.9 (214.3–241.5)	107,138	243.3 (228.4–258.3)	39,520	89.7 (83.6–95.9)	3,254	7.4 (7.0–7.8)	72,787	165.3 (154.2–176.4)	39,072	88.7 (67.9–109.6)
25–34	78,451	182.9 (171.8–194.0)	72,345	168.6 (158.5–178.8)	43,865	102.3 (96.0–108.5)	25,311	59.0 (53.6–64.4)	3,577	8.3 (8.0–8.7)	62,832	146.5 (136.0–157.0)	25,796	60.1 (45.5–74.8)
35–44	51,271	126.5 (118.4–134.5)	69,714	172.0 (162.5–181.4)	32,814	80.9 (76.0–85.9)	19,566	48.3 (44.1–52.5)	3,269	8.1 (7.8–8.4)	37,386	92.2 (85.7–98.8)	18,842	46.5 (35.7–57.3)
45–54	48,286	110.1 (103.5–116.7)	103,051	235.0 (222.8–247.2)	29,894	68.2 (64.0–72.4)	22,167	50.6 (46.2–54.9)	3,903	8.9 (8.7–9.1)	30,056	68.6 (63.6–73.5)	20,411	46.6 (35.7–57.4)
55–64	33,777	85.9 (80.7–91.0)	122,637	311.7 (296.8–326.7)	19,804	50.3 (47.1–53.6)	16,582	42.1 (37.5–46.8)	3,429	8.7 (8.5–8.9)	13,058	33.2 (30.9–35.5)	18,114	46.0 (36.3–55.8)
65–74	17,725	70.3 (65.9–74.6)	136,140	539.8 (511.7–567.9)	11,493	45.6 (42.4–48.8)	9,655	38.3 (33.4–43.2)	2,254	8.9 (8.8–9.1)	3,168	12.6 (11.4–13.7)	15,531	61.6 (50.7–72.5)
≥75	12,397	63.7 (59.3–68.1)	361,738	1,859.0 (1,747.4–1,970.7)	13,305	68.4 (62.6–74.1)	11,051	56.8 (48.3–65.3)	2,189	11.2 (11.0–11.5)	1,468	7.5 (6.7–8.4)	32,205	165.5 (137.1–193.9)
**Total**	**383,293**	**121.1 (114.4–127.9)**	**1,320,411**	**417.3 (395.9–438.6)**	**430,836**	**136.2 (127.5–144.8)**	**190,959**	**60.3 (55.3–65.4)**	**22,270**	**7.0 (6.9–7.2)**	**232,842**	**73.6 (68.9–78.3)**	**217,141**	**68.6 (53.2–84.1)**
**Adjusted** ^†††^	**121.7 (118.6–124.9)**		**413.2 (401.4–425.0)**		**142.1 (137.1–147.2)**		**61.3 (59.2–63.4)**		**6.8 (6.7–7.0)**		**75.5 (73.0–77.9)**		**68.9 (62.3–75.6)**	

**Sources: **For emergency department visits, Healthcare Cost and Utilization Project’s (HCUP) Nationwide Emergency Department Sample; for hospitalizations, HCUP’s National Sample; for deaths, CDC’s National Vital Statistics System.

**Abbreviations:** CI = confidence interval; TBI = traumatic brain injury.

*Visits with missing age were excluded. Numbers subject to rounding error.

^†^Persons who were hospitalized, died, or transferred to another facility were excluded.

^§^In-hospital deaths and patients who transferred from another hospital were excluded.

**^¶^**Record-Axis Condition codes were used (usually included both Part I and Part II of Entity-Axis Condition codes).

**Includes falls of undetermined intent to maintain consistency with past data releases.

^††^E-codes specify that the injury was unintentional but do not specify the actual mechanism of injury.

**^§§^**Includes TBIs in which the intent was not determined as well as those due to legal intervention or war. Includes TBIs in which no mechanism was specified in the record. Does not include falls of undetermined intent.

^¶¶^Age-adjusted to the 2000 U.S. standard population (per 100,000 population).

***Estimates suppressed for the age groups 0–4 and 5–14 years because it is unclear whether children aged <10 years are able to form suicidal intent.

^†††^Age-adjusted to the 2000 U.S. standard population (per 100,000 population).

### TBI-Related ED Visits, Hospitalizations, and Deaths by Sex and Principal Mechanism of Injury

Differences in age-adjusted rates were found for each principal mechanism when comparing rates of TBI-EDHDs by sex (Table 4). Overall, males had higher age-adjusted rates of TBI-EDHDs (959.0 per 100,000 population) compared with females (810.8). Males had higher age-adjusted rates of being struck by or against an object compared with females (167.3 versus 115.6), a higher age-adjusted rate of motor-vehicle crash–related TBI-EDHDs (128.2 versus 115.5), a more than fivefold higher age-adjusted rate of intentional self-harm-related TBI-EDHDs (11.7 versus 2.3), a higher age-adjusted rate of assault-related TBI-EDHDs (96.0 versus 54.8) (Table 4). Females had a significantly higher age-adjusted rate of fall-related TBI-EDHDs compared to men (417.7 versus 400.7).

**TABLE 4:** Estimated number* and age-adjusted rate of traumatic brain injury–related emergency department visits,^†^ hospitalizations,^§^ and deaths,^¶^ by sex and mechanism of injury — United States, 2013

Sex	Unintentional motor-vehicle crashes		Unintentional falls**		Unintentionally struck by or against an object		Other unintentional injury, mechanism unspecified^††^		Intentional self-harm		Assault or homicide		Other or no mechanism specified^§§^	
	No.	Rate^¶¶ ^(95% CI)	No.	Rate (95% CI)	No.	Rate (95% CI)	No.	Rate (95% CI)	No.	Rate (95% CI)	No.	Rate (95% CI)	No.	Rate (95% CI)
Male	201,012	128.2 (124.8–131.5)	603,444	400.7 (388.7–412.8)	253,848	167.3 (160.9–173.7)	119,637	77.4 (74.9–79.9)	18,509	11.7 (11.5–12.0)	149,271	96.0 (92.8–99.1)	118,904	77.7 (70.1–85.2)
Female	182,220	115.5 (112.3–118.8)***	716,915	417.7 (405.9–429.4)***	176,975	115.6 (111.7–119.5)***	71,306	45.1 (43.3–47.0)	3,760	2.3 (2.2–2.4)***	83,554	54.8 (52.7–57.0)***	98,225	59.8 (53.8–65.7)
**Total**	**383,231**	**121.7 (118.6–124.9)**	**1,320,360**	**413.2 (401.4–425.0)**	**430,822**	**142.1 (137.1–147.2)**	**190,944**	**61.3 (59.2–63.4)**	**22,270**	**6.8 (6.7–7.0)**	**232,828**	**75.4 (73.0–77.9)**	**217,129**	**68.9 (62.3–75.6)**

**Sources: **For emergency department visits, Healthcare Cost and Utilization Project’s (HCUP) Nationwide Emergency Department Sample; for hospitalizations, HCUP’s National Sample; for deaths, CDC’s National Vital Statistics System.

**Abbreviations**: CI = confidence interval; TBI = traumatic brain injury.

*Visits with missing age were excluded. Numbers subject to rounding error.

^†^Persons who were hospitalized, died, or transferred to another facility were excluded.

^§^In-hospital deaths and patients who transferred from another hospital were excluded.

**^¶^**Record-Axis Condition codes were used (usually included both Part I and Part II of Entity-Axis Condition codes).

**Includes falls of undetermined intent to maintain consistency with past data releases.

^††^E-codes specify that the injury was unintentional but do not specify the actual mechanism of injury.

^§§^Includes TBIs in which the intent was not determined as well as those due to legal intervention or war. Includes TBIs in which no mechanism was specified in the record. Does not include falls of undetermined intent.

^¶¶^Per 100,000 population.

***Significant difference in rate compared to men based on t-test (p<0.05). Only selected comparisons were tested for statistical significance.

### Comparison of TBI-Related ED Visits Between 2007 and 2013 by Age Group and Principal Mechanism of Injury

Age-adjusted rates of TBI-related ED visits increased from 534.4 per 100,000 in 2007 to 787.1 in 2013 (Table 2). Further, this increase in TBI-related ED visit rates has occurred for nearly all of the major principal mechanism categories: a 65% increase for falls (from 222.6 per 100,000 to 366.5, age-adjusted); a 55% increase in being struck by or against an object (from 90.3 to 139.8, age-adjusted); a 75% increase for TBIs as a result of intentional self-harm (from 0.4 to 0.7, age-adjusted); a 20% increase for assaults (from 56.8 to 68.2, age-adjusted); and a 19% increase for motor-vehicle crashes (from 83.8 to 99.5, age-adjusted) (Table 5).

**TABLE 5:** Estimated number* and rate of traumatic brain injury–related emergency department visits,^†^ by age group and mechanism of injury — Healthcare Cost and Utilization Project’s Nationwide Emergency Department Sample, United States, 2007 and 2013

Age group (yrs)	Year	Unintentional motor-vehicle crashes		Unintentional falls^§^		Unintentionally struck by or against an object		Other unintentional injury, mechanism unspecified^¶^		Intentional self-harm		Assault or homicide		Other or no mechanism specified**	
		No.	Rate^††^ (95% CI)	No.	Rate (95% CI)	No.	Rate (95% CI)	No.	Rate (95% CI)	No.	Rate (95% CI)	No.	Rate (95% CI)	No.	Rate (95% CI)
0–4	2007	5,582	26.7 (23.0–30.4)	165,093	789.1 (691.6–886.7)	38,850	185.7 (162.0–209.4)	10,317	49.3 (42.7–56.0)	**^§§^**	**^§§^**	624	3.0 (2.3–3.6)	13,528	64.7 (40.1–89.2)
	2013	5,251	26.4 (23.1–29.8)	217,280	1,094.4 (973.5–1,215.3)	51,517	259.5 (230.0–288.9)	12,592	63.4 (51.8–75.1)	**^§§^**	**^§§^**	590	3.0 (2.3–3.6)	18,696	94.2 (49.5–138.8)
5–14	2007	17,009	42.1 (37.7–46.6)	87,804	217.6 (193.6–241.5)	70,501	174.7 (156.6–192.8)	29,924	74.1 (66.9–81.4)	**^§§^**	**^§§^**	8,826	21.9 (19.4–24.3)	13,710	34.0 (22.2–45.8)
	2013	18,459	44.8 (40.1–49.4)	129,599	314.3 (282.0–346.6)	119,535	289.9 (259.9–319.9)	31,887	77.3 (69.6–85.0)	**^§§^**	**^§§^**	9,852	23.9 (21.1–26.7)	26,447	64.1 (39.1–89.2)
15–24	2007	88,161	205.1 (187.3–222.8)	64,574	150.2 (138.5–161.9)	70,128	163.1 (150.6–175.6)	29,338	68.2 (63.3–73.2)	427	1.0 (0.7–1.2)	63,320	147.3 (135.9–158.7)	24,899	57.9 (39.7–76.2)
	2013	97,214	220.8 (203.0–238.5)	96,202	218.5 (202.0–234.9)	106,066	240.9 (222.6–259.1)	36,272	82.4 (75.4–89.3)	808	1.8 (1.4–2.2)	68,210	154.9 (141.6–168.2)	36,417	82.7 (61.3–104.1)
25–34	2007	49,088	121.9 (110.2–133.7)	40,199	99.9 (92.0–107.7)	28,207	70.1 (64.5–75.7)	16,359	40.6 (37.3–43.9)	279	0.7 (0.5–0.9)	40,813	101.4 (92.3–110.5)	15,620	38.8 (28.1–49.6)
	2013	65,034	151.6 (138.6–164.6)	67,492	157.3 (145.1–169.6)	43,121	100.5 (92.8–108.2)	22,650	52.8 (46.9–58.7)	485	1.1 (0.8–1.4)	57,537	134.1 (122.0–146.3)	23,263	54.2 (39.2–69.2)
35–44	2007	35,299	82.7 (74.7–90.6)	41,668	97.6 (90.1–105.0)	23,222	54.4 (50.0–58.7)	14,435	33.8 (31.1–36.5)	264	0.6 (0.4–0.8)	29,200	68.4 (61.7–75.1)	12,676	29.7 (21.2–38.2)
	2013	42,402	104.6 (95.4–113.8)	64,113	158.1 (146.4–169.8)	32,203	79.4 (73.3–85.5)	17,156	42.3 (37.8–46.8)	392	1.0 (0.7–1.2)	33,568	82.8 (75.2–90.4)	16,795	41.4 (30.4–52.4)
45–54	2007	28,481	65.3 (59.0–71.7)	51,091	117.2 (108.0–126.4)	17,521	40.2 (36.9–43.5)	12,433	28.5 (26.2–30.8)	138	0.3 (0.2–0.4)	19,928	45.7 (41.0–50.5)	11,116	25.5 (18.8–32.2)
	2013	37,838	86.3 (78.8–93.8)	90,421	206.2 (190.9–221.5)	29,036	66.2 (61.0–71.4)	18,410	42.0 (37.3–46.7)	176	0.4 (0.3–0.5)	26,176	59.7 (54.0–65.4)	16,911	38.6 (27.6–49.6)
55–64	2007	15,797	48.4 (43.6–53.1)	46,823	143.4 (132.5–154.3)	8,739	26.8 (24.3–29.2)	6,680	20.5 (18.6–22.3)	**^¶¶^**	**^¶¶^**	5,136	15.7 (14.0–17.5)	6,873	21.0 (15.5–26.6)
	2013	25,477	64.8 (59.0–70.5)	103,924	264.2 (245.2–283.1)	19,007	48.3 (44.4–52.2)	13,130	33.4 (28.5–38.2)	135	0.3 (0.2–0.5)	10,828	27.5 (24.9–30.2)	13,684	34.8 (24.9–44.6)
65–74	2007	7,410	38.1 (34.3–41.9)	45,635	234.7 (216.3–253.0)	4,266	21.9 (19.6–24.3)	3,302	17.0 (15.3–18.7)	**^¶¶^**	**^¶¶^**	1,209	6.2 (5.3–7.2)	4,908	25.2 (18.6–31.8)
	2013	12,582	49.9 (45.4–54.4)	111,531	442.2 (408.6–475.8)	10,810	42.9 (39.3–46.5)	7,236	28.7 (23.7–33.6)	**^¶¶^**	**^¶¶^**	2,420	9.6 (8.4–10.8)	10,052	39.9 (29.0–50.7)
≥75	2007	5,631	30.5 (27.2–33.7)	129,618	701.5 (638.0–764.9)	5,740	31.1 (27.6–34.6)	3,847	20.8 (18.1–23.5)	**^¶¶^**	**^¶¶^**	582	3.1 (2.5–3.8)	10,132	54.8 (40.9–68.8)
	2013	7,791	40.0 (35.8–44.3)	282,912	1,453.9 (1,327.1–1,580.8)	12,182	62.6 (56.3–68.9)	7,775	40.0 (31.4–48.5)	**^¶¶^**	**^¶¶^**	1,040	5.3 (4.5–6.2)	19,403	99.7 (71.5–128.0)
**Total**	**2007**	**252,459**	**83.8 (76.5–91.1)**	**672,504**	**223.1 (206.1–240.2)**	**267,175**	**88.6 (81.7–95.6)**	**126,635**	**42.0 (39.1–44.9)**	**1,254**	**0.4 (0.3–0.5)**	**169,638**	**56.3 (51.6–60.9)**	**113,460**	**37.6 (27.0–48.3)**
	**2013**	**312,048**	**98.6 (90.7–106.6)**	**1,163,473**	**367.7 (341.2–394.1)**	**423,477**	**133.8 (123.4–144.2)**	**167,107**	**52.8 (47.3–58.4)**	**2,284**	**0.7 (0.6–0.8)**	**210,220**	**66.4 (60.8–72.1)**	**181,668**	**57.4 (41.7–73.1)**
Adjusted***	**2007**	**83.8 (80.3–87.2)**		**222.6 (213.6–231.6)**		**90.3 (86.6–94.0)**		**42.7 (41.1–44.2)**		**0.4 (0.4–0.5)**		**56.8 (54.4–59.2)**		**37.8 (33.5–42.0)**	
	**2013**	**99.5 (95.9–103.2)** ^†††^		**366.5 (353.4–379.7)** ^†††^		**139.8 (134.1–145.5)** ^†††^		**54.0 (51.8–56.2)**		**0.7 (0.7–0.8)** ^†††^		**68.2 (65.3–71.1)** ^†††^		**58.3 (51.6–65.0)**	

**Abbreviations: **CI = confidence interval; TBI = traumatic brain injury.

*Visits with missing age were excluded. Numbers subject to rounding error.

^†^Persons who were hospitalized, died, or transferred to another facility were excluded.

^§^Includes falls of undetermined intent to maintain consistency with past data releases.

^¶^E-codes specify that the injury was unintentional but do not specify the actual mechanism of injury.

**Includes TBIs in which the intent was not determined as well as those due to legal intervention or war. Includes TBIs in which no mechanism was specified in the record. Does not include falls of undetermined intent.

^††^Per 100,000 population.

^§§^Estimates suppressed for the age groups 0–4 and 5–14 years because it is unclear whether children aged <10 years are able to form suicidal intent.

**^¶¶^**The relative standard error was >30% or the standard error = 0, the value of the estimate was considered unreliable and was not reported.

***Age-adjusted to the 2000 U.S. standard population (per 100,000 population).

^†††^Significant difference in rate compared to 2007 based on t-test (p<0.05). Only selected comparisons were tested for statistical significance.

The number of TBI-related ED visits increased from approximately 1.6 million in 2007 to approximately 2.5 million in 2013, representing an increase of more than 50%, or an increase of more than 850,000 ED visits (Table 2). The key contributors to the increase are falls (accounting for 57.3% of the increase); being struck by or against an object (accounting for 18.2% of the increase); and motor-vehicle crashes (accounting for 7.0% of the increase). With respect to both principal mechanism and age, the key contributors to the increase are fall-related TBIs among those aged ≥75 years (accounting for 17.9% of the increase), aged 65–74 years (accounting for 7.7% of the increase), aged 55–64 years (accounting for 6.7% of the increase), and aged 0–4 years (accounting for 6.1% of the increase) and being struck by or against an object among those aged 5–14 years (accounting for 5.7% of the increase) and 15–24 years (accounting for 4.2% of the increase).

### Comparison of TBI-Related Hospitalizations Between 2007 and 2013 by Age Group and Principal Mechanism of Injury

Approximately 70% of TBI-related hospitalizations in 2013 were attributable to two principal mechanisms: falls (50.4%) and motor-vehicle crashes (21.5%) (Table 6). Overall, the total number of TBI-related hospitalizations in 2013 (281,555) was similar to the number of TBI-related hospitalizations in 2007 (267,350) and rates of TBI-related hospitalizations remained nearly the same (Table 2). The age-adjusted rate of TBI-related hospitalizations attributable to motor-vehicle crashes decreased from 2007 to 2013 (from 23.5 to 18.8 per 100,000) (Table 6). In addition, the age-adjusted rate of TBI-related hospitalizations attributable to falls increased from 33.9 in 2007 to 42.2 in 2013.

**TABLE 6:** Estimated number* and rate of traumatic brain injury–related hospitalizations,^†^ by age group and mechanism of injury — Healthcare Cost and Utilization Project’s National Sample, United States, 2007 and 2013

Age group (yrs)	Year	Unintentional motor-vehicle crashes		Unintentional falls^§^		Unintentionally struck by or against an object		Other unintentional injury, mechanism unspecified^¶^		Intentional self-harm		Assault or homicide		Other or no mechanism specified**	
		No.	Rate^††^ (95% CI)	No.	Rate (95% CI)	No.	Rate (95% CI)	No.	Rate (95% CI)	No.	Rate (95% CI)	No.	Rate (95% CI)	No.	Rate (95% CI)
0–4	2007	1,345	6.4 (4.1–8.8)	5,333	25.5 (19.3–31.7)	610	2.9 (1.9–3.9)	657	3.1 (2.1–4.2)	^§§^	^§§^	1,166	5.6 (3.4–7.8)	1,819	8.7 (4.5–12.9)
	2013	880	4.4 (3.5–5.4)	4,925	24.8 (21.3–28.3)	600	3.0 (2.3–3.7)	765	3.9 (3.0–4.7)	^§§^	^§§^	1,065	5.4 (4.2–6.5)	1,015	5.1 (3.5–6.7)
5–14	2007	3,917	9.7 (6.9–12.5)	2,823	7.0 (5.4–8.6)	1,278	3.2 (2.4–4.0)	2,515	6.2 (4.7–7.8)	^§§^	^§§^	302	0.7 (0.5–1.0)	1,608	4.0 (1.8–6.2)
	2013	2,515	6.1 (5.1–7.1)	2,585	6.3 (5.3–7.2)	825	2.0 (1.6–2.4)	1,615	3.9 (3.2–4.6)	^§§^	^§§^	155	0.4 (0.2–0.5)	955	2.3 (1.6–3.1)
15–24	2007	20,318	47.3 (37.9–56.6)	4,522	10.5 (8.5–12.6)	1,540	3.6 (2.9–4.2)	4,009	9.3 (7.7–11.0)	277	0.6 (0.4–0.9)	5,120	11.9 (9.8–14.0)	5,075	11.8 (6.4–17.2)
	2013	14,025	31.8 (28.6–35.1)^***^	4,035	9.2 (8.3–10.0)	1,045	2.4 (2.0–2.7)	2,950	6.7 (5.9–7.5)	365	0.8 (0.6–1.0)	3,360	7.6 (6.7–8.5)	2,550	5.8 (4.6–7.0)
25–34	2007	11,760	29.2 (23.3–35.1)	3,697	9.2 (7.7–10.7)	842	2.1 (1.7–2.5)	2,364	5.9 (4.9–6.9)	252	0.6 (0.4–0.8)	4,657	11.6 (9.5–13.6)	4,494	11.2 (6.7–15.7)
	2013	11,400	26.6 (23.8–29.3)	4,680	10.9 (10.0–11.9)	705	1.6 (1.4–1.9)	2,315	5.4 (4.7–6.0)	390	0.9 (0.7–1.1)	4,130	9.6 (8.6–10.7)	2,370	5.5 (4.4–6.7)
35–44	2007	9,962	23.3 (18.3–28.4)	5,756	13.5 (11.6–15.4)	781	1.8 (1.5–2.2)	2,495	5.8 (4.9–6.8)	253	0.6 (0.4–0.8)	3,860	9.0 (7.3–10.8)	3,967	9.3 (5.6–13.0)
	2013	7,465	18.4 (16.4–20.4)	5,280	13.0 (12.0–14.1)	570	1.4 (1.1–1.7)	2,040	5.0 (4.4–5.6)	265	0.7 (0.5–0.8)	3,035	7.5 (6.6–8.3)	1,890	4.7 (3.8–5.5)
45–54	2007	9,895	22.7 (18.0–27.4)	9,349	21.5 (18.8–24.1)	788	1.8 (1.5–2.2)	2,835	6.5 (5.4–7.6)	259	0.6 (0.4–0.8)	3,561	8.2 (6.6–9.8)	5,109	11.7 (8.1–15.3)
	2013	8,925	20.4 (18.3–22.5)	11,725	26.7 (25.0–28.4)	805	1.8 (1.5–2.1)	3,145	7.2 (6.5–7.9)	320	0.7 (0.5–0.9)	3,230	7.4 (6.6–8.2)	3,210	7.3 (6.2–8.4)
55–64	2007	6,453	19.8 (16.0–23.5)	10,686	32.7 (29.3–36.2)	647	2.0 (1.6–2.4)	2,117	6.5 (5.4–7.6)	97	0.3 (0.2–0.4)	1,311	4.0 (3.1–4.9)	4,279	13.1 (9.5–16.7)
	2013	7,040	17.9 (16.0–19.8)	17,205	43.7 (41.3–46.1)	735	1.9 (1.6–2.2)	2,875	7.3 (6.6–8.0)	150	0.4 (0.2–0.5)	1,770	4.5 (3.9–5.1)	4,040	10.3 (9.0–11.5)
65–74	2007	3,562	18.3 (14.9–21.7)	14,600	75.1 (68.0–82.2)	419	2.2 (1.6–2.7)	1,144	5.9 (4.9–6.9)	^¶¶^	^¶¶^	348	1.8 (1.2–2.4)	4,464	23.0 (18.4–27.5)
	2013	4,395	17.4 (15.6–19.2)	22,390	88.8 (84.5–93.0)	650	2.6 (2.1–3.0)	1,985	7.9 (7.0–8.8)	75	0.3 (0.2–0.4)	540	2.1 (1.7–2.6)	5,125	20.3 (18.2–22.4)
≥75	2007	4,063	22.0 (18.4–25.6)	47,553	257.3 (235.4–279.3)	779	4.2 (3.4–5.0)	1,618	8.8 (7.5–10.0)	57	0.3 (0.1–0.5)	322	1.7 (1.3–2.2)	11,563	62.6 (52.7–72.5)
	2013	3,820	19.6 (17.7–21.6)	69,030	354.8 (340.1–369.4)***	1,075	5.5 (4.7–6.3)	2,390	12.3 (11.0–13.5)	70	0.4 (0.2–0.5)	235	1.2 (0.9–1.6)	11,805	60.7 (54.6–66.7)
**Total**	**2007**	**71,274**	**23.6 (19.2–28.1)**	**104,319**	**34.6 (31.5–37.7)**	**7,684**	**2.5 (2.2–2.9)**	**19,754**	**6.6 (5.7–7.5)**	**1,293**	**0.4 (0.3–0.5)**	**20,648**	**6.9 (5.7–8.0)**	**42,378**	**14.1 (10.2–18.0)**
	**2013**	**60,465**	**19.1 (17.4–20.8)**	**141,855**	**44.8 (43.1–46.6)**	**7,010**	**2.2 (2.1–2.4)**	**20,080**	**6.3 (5.9–6.8)**	**1,665**	**0.5 (0.5–0.6)**	**17,520**	**5.5 (5.1–6.0)**	**32,960**	**10.4 (9.3–11.5)**
**Adjusted^†††^**	**2007**	**23.5 (21.5–25.4)**		**33.9 (32.3–35.5)**		**2.6 (2.4–2.8)**		**6.5 (6.1–7.0)**		**0.4 (0.4–0.5)**			**6.9 (6.3–7.4)**	**13.9 (12.4–15.4)**	
	**2013**	18.8 (18.1–19.6)***		42.2 (41.1–43.2)***		**2.2 (2.1–2.3)**		**6.2 (5.9–6.4)**		**0.5 (0.5–0.6)**		**5.6 (5.3–5.9)**		**9.9 (9.4–10.4)**	

**Abbreviations:** CI = confidence interval; TBI = traumatic brain injury.

*Visits with missing age were excluded. Numbers subject to rounding error.

^†^In-hospital deaths and patients who transferred from another hospital were excluded.

^§^Includes falls of undetermined intent to maintain consistency with past data releases.

^¶^E-codes specify that the injury was unintentional but do not specify the actual mechanism of injury.

**Includes TBIs in which the intent was not determined as well as those due to legal intervention or war. Includes TBIs in which no mechanism was specified in the record. Does not include falls of undetermined intent.

^††^Per 100,000 population.

^§§^Estimates suppressed for the age groups 0–4 and 5–14 years because it is unclear whether children aged <10 years are able to form suicidal intent.

^¶¶^The relative standard error was >30% or the standard error = 0, the value of the estimate was considered unreliable and was not reported.

***Significant difference in rate compared to 2007 based on t-test (p< .05). Only selected comparisons were tested for statistical significance.

^†††^Age-adjusted to the 2000 U.S. standard population (per 100,000 population).

Age was an important factor contributing to the TBI-related hospitalization rate change from 2007 to 2013. The overall decrease in rates of TBI-related hospitalizations attributable to motor-vehicle crashes was evidenced most prominently among those aged 15–24 years as the rate decreased from 47.3 in 2007 to 31.8 in 2013 (Table 6). The overall increase in rates of TBI-related hospitalizations attributable to falls was observed most prominently among those aged ≥75 years (from 257.3 in 2007 to 354.8 in 2013).

### Comparison of TBI-Related Deaths Between 2007 and 2013 by Age Group and Principal Mechanism of Injury

Overall, the number of TBI-related deaths increased from 54,699 in 2007 to 55,920 in 2013 (Table 2). However, age-adjusted rates of TBI-related deaths decreased slightly during that period (from 17.9 to 17.0 per 100,000). This decrease is largely attributable to an overall decrease in the age-adjusted rate of TBI-related deaths attributable to motor-vehicle crashes (5.0 in 2007 to 3.4 in 2013) (Table 7). Despite the overall decrease in rates, there were increases in the age-adjusted rate of TBI-related deaths attributable to falls (from 3.8 in 2007 to 4.5 in 2013) and intentional self-harm (from 4.8 in 2007 to 5.6 in 2013). Whereas motor-vehicle crashes were the leading cause of TBI-related death in 2007, in both number and rate, intentional self-harm was the leading cause, in number and rate, in 2013. Increases between 2007 and 2013 in the number of TBI-related deaths attributable to self-harm were found among all age groups examined (i.e., those aged ≥15 years). In 2013, of TBI-related deaths attributable to self-harm, 86.9% were among males; in 96.9% of these cases, a firearm was the principal mechanism (data not shown). Although leading causes of TBI-related deaths for all other age groups are either falls, motor-vehicle crashes, or unintentional self-harm, the leading cause among those aged 0–4 years in 2007 and 2013 was assault/homicide (Table 7).

**TABLE 7:** Number* and rate of traumatic brain injury-related deaths,^†^ by age group and mechanism of injury — CDC’s National Vital Statistics System, United States, 2007 and 2013

Age group (yrs)	Year	Unintentional motor-vehicle crashes		Unintentional falls^§^		Unintentionally struck by or against an object		Other unintentional injury, mechanism unspecified^¶^		Intentional self-harm		Assault or homicide		Other or no mechanism specified**	
		No.	Rate^††^ (95% CI)	No.	Rate (95% CI)	No.	Rate (95% CI)	No.	Rate (95% CI)	No.	Rate (95% CI)	No.	Rate (95% CI)	No.	Rate (95% CI)
0–4	2007	317	1.5 (1.3–1.7)	41	0.2 (0.1–0.3)	35	0.2 (0.1–0.2)	150	0.7 (0.6–0.8)	—^¶¶^	—	402	1.9 (1.7–2.1)	33	0.2 (0.1–0.2)
	2013	204	1.0 (0.9–1.2)^§§^	28	0.1 (0.1–0.2)	31	0.2 (0.1–0.2)	128	0.6 (0.5–0.8)	—	—	324	1.6 (1.5–1.8)	45	0.2 (0.2–0.3)
5–14	2007	576	1.4 (1.3–1.5)	16	0 (0–0.1)	22	0.1 (0–0.1)	170	0.4 (0.4–0.5)	—	—	130	0.3 (0.3–0.4)	16	0 (0–0.1)
	2013	334	0.8 (0.7–0.9)^§§^	6	0 (0–0)	15	0 (0–0.1)	121	0.3 (0.2–0.3)	—	—	102	0.2 (0.2–0.3)	12	0 (0–0)
15–24	2007	4,287	10.0 (9.7–10.3)	144	0.3 (0.3–0.4)	30	0.1 (0–0.1)	480	1.1 (1.0–1.2)	1,794	4.2 (4.0–4.4)	1,686	3.9 (3.7–4.1)	129	0.3 (0.2–0.4)
	2013	2,504	5.7 (5.5–5.9)^§§^	127	0.3 (0.2–0.3)	27	0.1 (0–0.1)	298	0.7 (0.6–0.8)	2,081	4.7 (4.5–4.9)^§§^	1,217	2.8 (2.6–2.9)	105	0.2 (0.2–0.3)
25–34	2007	2,611	6.5 (6.2–6.7)	201	0.5 (0.4–0.6)	45	0.1 (0.1–0.1)	431	1.1 (1.0–1.2)	2,092	5.2 (5.0–5.4)	1,362	3.4 (3.2–3.6)	120	0.3 (0.2–0.4)
	2013	2,017	4.7 (4.5–4.9)^§§^	173	0.4 (0.3–0.5)	39	0.1 (0.1–0.1)	346	0.8 (0.7–0.9)	2,702	6.3 (6.1–6.5)^§§^	1,165	2.7 (2.6–2.9)	163	0.4 (0.3–0.4)
35–44	2007	2,114	5.0 (4.7–5.2)	371	0.9 (0.8–1.0)	47	0.1 (0.1–0.1)	514	1.2 (1.1–1.3)	2,462	5.8 (5.5–6.0)	960	2.2 (2.1–2.4)	182	0.4 (0.4–0.5)
	2013	1,404	3.5 (3.3–3.6)^§§^	321	0.8 (0.7–0.9)	41	0.1 (0.1–0.1)	370	0.9 (0.8–1.0)	2,612	6.4 (6.2–6.7)^§§^	783	1.9 (1.8–2.1)	157	0.4 (0.3–0.4)
45–54	2007	2,127	4.9 (4.7–5.1)	875	2.0 (1.9–2.1)	67	0.2 (0.1–0.2)	724	1.7 (1.5–1.8)	2,923	6.7 (6.5–6.9)	765	1.8 (1.6–1.9)	293	0.7 (0.6–0.7)
	2013	1,523	3.5 (3.3–3.6)^§§^	905	2.1 (1.9–2.2)	53	0.1 (0.1–0.2)	612	1.4 (1.3–1.5)	3,407	7.8 (7.5–8.0)^§§^	650	1.5 (1.4–1.6)	290	0.7 (0.6–0.7)
55–64	2007	1,279	3.9 (3.7–4.1)	1,139	3.5 (3.3–3.7)	46	0.1 (0.1–0.2)	497	1.5 (1.4–1.7)	2,281	7.0 (6.7–7.3)	405	1.2 (1.1–1.4)	270	0.8 (0.7–0.9)
	2013	1,260	3.2 (3.0–3.4)^§§^	1,508	3.8 (3.6–4.0)	62	0.2 (0.1–0.2)	577	1.5 (1.3–1.6)	3,144	8.0 (7.7–8.3)^§§^	460	1.2 (1.1–1.3)	390	1.0 (0.9–1.1)
65–74	2007	775	4.0 (3.7–4.3)	1,650	8.5 (8.1–8.9)	44	0.2 (0.2–0.3)	415	2.1 (1.9–2.3)	1,408	7.2 (6.9–7.6)	171	0.9 (0.7–1.0)	279	1.4 (1.3–1.6)
	2013	748	3.0 (2.8–3.2)^§§^	2,219	8.8 (8.4–9.2)	33	0.1 (0.1–0.2)	434	1.7 (1.6–1.9)	2,141	8.5 (8.1–8.8)^§§^	208	0.8 (0.7–0.9)	354	1.4 (1.3–1.5)
≥75	2007	994	5.4 (5.0–5.7)	7,344	39.7 (38.8–40.7)	40	0.2 (0.1–0.3)	990	5.4 (5.0–5.7)	1,828	9.9 (9.4–10.3)	177	1.0 (0.8–1.1)	878	4.8 (4.4–5.1)
	2013	786	4.0 (3.8–4.3)^§§^	9,796	50.3 (49.3–51.3)^§§^	48	0.2 (0.2–0.3)	886	4.6 (4.3–4.9)	2,100	10.8 (10.3–11.3)^§§^	193	1.0 (0.9–1.1)	997	5.1 (4.8–5.4)
**Total**	**2007**	**15,080**	**5.0 (4.9–5.1)**	**11,781**	**3.9 (3.8–4.0)**	**376**	**0.1 (0.1–0.1)**	**4,371**	**1.5 (1.4–1.5)**	**14,833**	**4.9 (4.8–5.0)**	**6,058**	**2.0 (2.0–2.1)**	**2,200**	**0.7 (0.7–0.8)**
	**2013**	**10,780**	**3.4 (3.3–3.5)** ^§§^	**15,083**	**4.8 (4.7–4.8)**	**349**	**0.1 (0.1–0.1)**	**3,772**	**1.2 (1.2–1.2)**	**18,321**	**5.8 (5.7–5.9)**	**5,102**	**1.6 (1.6–1.7)**	**2,513**	**0.8 (0.8–0.8)**
Adjusted***	**2007**	**5.0 (4.9–5.0)**		**3.8 (3.7–3.9)**		**0.1 (0.1–0.1)**		**1.4 (1.4–1.5)**		**4.8 (4.7–4.9)**		**2.0 (2.0–2.1)**		**0.7 (0.7–0.7)**	
	**2013**	**3.4 (3.3–3.4)^§§^**		**4.5 (4.4–4.5)^§§^**		**0.1 (0.1–0.1)**		**1.1 (1.1–1.2)**		**5.6 (5.5–5.6)^§§^**		**1.6 (1.6–1.7)**		**0.7 (0.7–0.8)**	

**Abbreviations: **CI = confidence interval; TBI = traumatic brain injury.

*Visits with missing age were excluded. Numbers subject to rounding error.

^†^Record-Axis Condition codes were used (usually included both Part I and Part II of Entity-Axis Condition codes).

^§^Includes falls of undetermined intent to maintain consistency with past data releases.

^¶^E-codes specify that the injury was unintentional but do not specify the actual mechanism of injury.

**Includes TBIs in which the intent was not determined as well as those due to legal intervention or war. Includes TBIs in which no mechanism was specified in the record. Does not include falls of undetermined intent.

^††^Per 100,000 population.

^§§^Significant difference in rate compared to 2007 based on t-test (p<0.05). Only selected comparisons were tested for statistical significance.

^¶¶^Estimates suppressed for the age groups 0–4 and 5–14 years because it is unclear whether children aged <10 years are able to form suicidal intent.

***Age-adjusted to the 2000 U.S. standard population (per 100,000 population).

The overall decrease in the rate of TBI-related deaths attributable to motor-vehicle crashes was found among all age groups, with the most pronounced decrease being among those aged 15–24 years (from 10.0 in 2007 to 5.7 in 2013). The increased rate of TBI-related deaths attributable to falls was not evenly spread among all ages as those aged ≥75 years had the largest increase (from 39.7 in 2007 to 50.3 in 2013).

## Discussion

In 2013, approximately 2.8 million TBI-EDHDs occurred in the United States. Most were TBI-related ED visits (87.9%), and only 2.0% of the total were TBI-related deaths. Males continue to have higher rates of TBI-EDHDs compared with females (*1*,*14*). Although the total number of TBI-EDHDs has increased over time, the increases are not uniform across all age groups or principal mechanisms of injury, and this suggests priority areas for TBI-related prevention.

Several hypotheses might explain the increase in TBI-EDHDs over time. First, heightened public awareness about sports-related concussions might have translated to greater public concern about the effects of TBI generally, leading people of all ages to more readily seek care. Second, heightened awareness among health care providers, and the broader dissemination of validated assessment tools, might have resulted in more TBI diagnoses. Although increases among youth were found for TBI-related ED visits, there were significant increases in the number of ED visits, hospitalizations, and deaths attributable to TBIs resulting from older adult falls. This across-the-board increase over a relatively short time, suggests the need to address preventing and reducing the number of older adult falls resulting in TBI.

The highest rates of TBI-EDHDs were among the oldest or youngest age groups. TBIs in these age groups are notable for several reasons. In children aged <7 years, TBIs can impair neurologic development and the ability to meet developmental milestones (*15*). Impaired development might lead to further challenges as a child ages, such as declines in academic functioning and psychosocial sequelae such as emotional and behavioral disorders (e.g., depression or attention-deficit hyperactivity disorder) (*16*–*19*). In older adults, TBIs are associated more often with hospitalization and death. Cognitive and physical reserve are diminished at older ages, so TBIs might have a greater impact on daily living. TBIs in older adults are more likely to lead to hospitalization and these hospitalizations can be complicated by the presence of comorbidities. Furthermore, more frequent use of anticoagulants among older adults can result in a greater likelihood of secondary effects because of an increased likelihood of intracranial hemorrhage (*20*,*21*).

The most common principal mechanisms of injury for TBI-EDHDs were falls, being struck by or against an object, and motor-vehicle crashes. Although these three principal mechanisms accounted for approximately 70% of all TBI-EDHDs, particular age groups were disproportionally affected by specific principal mechanisms, similar to previous studies (*1*,*14*,*22*,*23*). Approximately half of all fall-related TBI-EDHDs occurred among those aged 0–4 years and ≥75 years. Codes defining whether a TBI was fall-related are heterogeneous. In addition to falls attributable to tripping and slipping, the codes also capture falls on stairs or from ladders, falls from one level to another (e.g., from a bed or chair), and falls into openings such as swimming pools. This analysis did not examine the individual contribution of each fall-related code. Future analyses could examine these individual codes to delineate better how activities leading to fall-related TBI vary by age group.

Certain prevention strategies have been identified for older adult falls, many of which have been demonstrated in randomized controlled trials to be effective (*24*). These include multicomponent physical exercise programs, Tai chi, Vitamin D supplementation (which might be effective among those who are Vitamin D deficient), surgical interventions (e.g., pacemakers and cataract surgery where indicated), and strategies to reduce home hazards (e.g., increased lighting and removal of tripping hazards). CDC has developed the STEADI initiative (Stopping Elderly Accidents Deaths and Injuries) as a comprehensive strategy to reduce falls in older adults. STEADI incorporates empirically supported clinical guidelines and scientifically tested interventions to help primary care providers address patient fall risk through the identification of modifiable risk factors and implementation of effective interventions (e.g., medication management) (*25*).

A number of strategies have been suggested for preventing injuries in children in general, including those resulting from falls. These include the use of safety gates at the top and bottom of stairways; ensuring that children aged <6 years do not sleep in the top bunk of a bunk bed; seat belt use in a shopping cart; use of an appropriate helmet for activities such as bicycle riding, skateboarding, and horseback riding; and age- and activity-appropriate supervision by adult caregivers (*26*–*28*). To prevent TBIs related to motor-vehicle crashes among infants and children, children should sit in the back seat until aged 13 years and be seated in age- and size-appropriate car seats (*29*). Unrestrained children aged 4–15 years are three times more likely to sustain a TBI than children who were restrained (*30*). The American Academy of Pediatrics (AAP) recommends that infants and toddlers remain in a rear-facing car safety seat until aged 2 years or until they reach the height and weight limit designated by the manufacturer of the car seat, then be restrained in forward-facing car seats (until they reach the height/weight limits on their car seats) (*29*). AAP recommends that children sit in a booster seat once they outgrow child safety seats and can fit appropriately into an adult seat belt (*29*).

The leading cause of TBI-related death among those aged 0–4 years was assault/homicide, including abusive head trauma by inflicted blunt impact or violent shaking, and other causes, such as firearm-related injuries (*31*). A number of strategies have been developed to prevent child abuse and neglect. To help communities make use of the best available evidence for prevention, CDC has released a technical package that describes a set of strategies and examples of specific approaches that enhance safe, stable, nurturing relationships and environments for children and families to reduce abuse and neglect and promote health (*32*).

The highest rates of TBI-EDHDs occurring after being struck by or against objects were among those aged 0–24 years. Sports- and recreation-related activities likely contribute to these types of injuries, especially for those aged 4–24 years. However, this analysis did not look at specific E-codes as a means to identify sports- and recreation-related TBIs. Although E-codes have been used to identify sports-related injuries (*33*), it is challenging to describe all activities associated with sports and recreation using these data due to differences in coding policies among health care providers, limited documentation in medical records, and limited utility of E-codes. To obtain greater detail about the nature of “struck by or against” related injuries and identify targets for prevention, use of other data sources would be needed. For example, the National Electronic Injury Surveillance System–All Injury Program (NEISS-AIP) includes narratives with each record that provide a short description of the injury event. Sometimes this includes what the person was doing at the time of injury and additional parties involved. Such information has been more useful in identifying risk factors for strike-related TBI incidence (*22*).

The most common principal mechanism of TBI-EDHDs among persons aged 15–24 and 25–34 years was motor-vehicle crashes. This corresponds with the age groups that are known to be at higher risk for a motor-vehicle crash (*34*). Several factors have contributed to increased motor vehicle-related injuries in teens and young adult drivers compared to older drivers, including a reduced ability to recognize driving hazards and unsafe road conditions (*35*), low frequency of seat belt use (*36*), and higher levels of alcohol-impaired driving (*37*). The motor vehicle mechanism category used in the analysis includes injuries to pedal cyclists and pedestrians by motor vehicles. However, previous research has shown that approximately 70% of the motor-vehicle–related TBIs in persons aged 15–34 years involve occupants of a vehicle, approximately 12% involve motorcycle passengers, and approximately 8% involve pedestrians (*1*).

Compared to 2007, in 2013 the overall age-adjusted rate of TBI-related hospitalizations attributable to motor-vehicle crashes decreased, with the largest decreases occurring among those aged 15–24 years. This may be due to the implementation of programs and policies focused on young and inexperienced drivers. Programs such as graduated drivers licensing focus on young drivers as a way to increase safety awareness and reduce driving under high-risk driving conditions when the driver is still relatively inexperienced (*38*). All states have instituted zero tolerance for alcohol and driving among young drivers (i.e., those aged <20 years) (*39*). Overall decreases in these motor-vehicle–related hospitalizations were likely the result of both behavioral and vehicle improvements such as the increased use of seatbelts and vehicle safety measures such as airbags and electronic stability control (*40*–*42*). These advances have helped to reduce the incidence of motor-vehicle crashes but might also contribute to decreased injury severity, resulting in fewer TBIs that require hospitalization.

Although the number of TBI-related deaths increased from 2007 to 2013, the age-adjusted rate of TBI-related deaths decreased. This decrease was largely attributable to a decrease in the rate of fatal TBIs in motor-vehicle crashes. However, to some extent, this decrease has masked increases during the same period in fatal TBIs caused by falls, particularly among older adults, and an increase in fatal TBIs attributable to intentional self-harm. Fatal TBIs attributable to intentional self-harm were found predominantly among males; firearms were the predominant principal mechanism of injury. The increase in fatal TBIs attributable to intentional self-harm is consistent with an overall increase in suicide rates in the United States and underscores the importance of comprehensive and coordinated prevention efforts, including efforts to enhance social support and connectedness, reduce stigma for help-seeking, and provide support for those at greatest risk (*43**,**44*).

The increase in the overall number of TBI-related hospitalizations and deaths attributable to older adult falls might be in some part attributable to increased life expectancy combined with the increased risk of falls among older adults (*45*). However, the increase in life expectancy during the relatively short period of time covered by this analysis (from 78.1 years in 2007 to 78.8 in 2013) cannot explain such a large increase in the rate of TBI-related hospitalizations and deaths attributable to falls among older adult age groups. The reason or reasons for this increase are unknown.

## Limitations

The findings in this report are subject to at least three limitations. First, these findings might overestimate the incidence of TBI-related ED visits and hospitalizations because the data are collected at the visit-level only. Thereby multiple records might exist for a single injury as persons might present more than once for treatment. However, steps were taken to reduce the number of records that were double-counted, including removing in-hospital deaths (that would be included in the mortality data) and excluding hospital transfers but residual double counting for multiple encounters/readmissions cannot be ruled out. Second, clinical severity cannot be ascertained from hospital administrative data that are not primarily designed for public health surveillance. Although TBIs are included as a diagnosis in the HCUP NIS and NEDS records, the severity of these injuries is unknown, nor is it known if hospital admissions with multiple diagnoses were attributable to the head injury or to another diagnosis. Third, this study does not include data on persons who did not seek care for their TBI or those who sought care in an outpatient setting. As a result, the TBI-EDHDs described in this report are not a complete accounting of all TBIs occurring in the United States and likely an undercount of TBIs that are milder in severity.

## Conclusion

In 2013, approximately 2.8 million TBI-related ED visits, hospitalizations, and deaths occurred in the United States, representing an increase since 2007 that was largely attributed to an increase in the number and rate of TBI-related ED visits. Although much public interest has been devoted to sports-related concussion in youth, the findings in this report indicate that older adult falls account for a much larger proportion of the increase in TBI-related ED visits during this period. In addition, although the modest increases in ED visits that might be attributed to youth sports concussion do not extend to increases in TBI-related hospitalizations and deaths, the same cannot be said for TBIs attributable to older adult falls. From 2007 to 2013, increases in TBI-related hospitalizations and deaths attributable to older adult falls suggest the need for greater attention to preventing older adult falls. Empirically validated prevention measures can help reduce the incidence of older adult falls.

The decrease in TBI-related deaths caused by motor-vehicle crashes from 2007 to 2013 is likely attributable to efforts to prevent motor-vehicle crashes. Nevertheless, more can be done to further reduce motor-vehicle crashes. The United States lags behind other high-income countries in the rate of motor-vehicle crash deaths; in 2013, the rate of motor-vehicle crash deaths in the United States was more than double the average rate of other high-income comparison countries (*46*). Redoubling efforts to increase restraint use and reduce alcohol-impaired driving, among other proven interventions, are critical to the continued reduction of motor-vehicle crashes and motor-vehicle crash–related TBIs.
